# Leiomyoma with KAT6B-KANSL1 fusion: case report of a rapidly enlarging uterine mass in a postmenopausal woman

**DOI:** 10.1186/s13000-019-0809-1

**Published:** 2019-04-25

**Authors:** Alessandra J. Ainsworth, Nooshin K. Dashti, Taofic Mounajjed, Karen J. Fritchie, Jaime Davila, Rohini Mopuri, Rory A. Jackson, Kevin C. Halling, Jamie N. Bakkum-Gamez, J. Kenneth Schoolmeester

**Affiliations:** 1 Department of Obstetrics and Gynecology, Mayo Clinic, 200 First Street SW, Rochester, MN 55905 USA; 2 Department of Laboratory Medicine and Pathology, Mayo Clinic, Rochester, MN USA

**Keywords:** KAT6B-KANSL1, Leiomyoma, Uterus

## Abstract

**Background:**

Uterine leiomyomas, in contrast to sarcomas, tend to cease growth following menopause. In the setting of a rapidly enlarging uterine mass in a postmenopausal patient, clinical distinction of uterine leiomyoma from sarcoma is difficult and requires pathologic examination.

**Case presentation:**

A 74-year-old woman presented with postmenopausal bleeding and acute blood loss requiring transfusion. She was found to have a rapidly enlarging uterine mass clinically suspicious for sarcoma. An abdominal hysterectomy and bilateral salpingo-oophorectomy were performed. A 15.5 cm partially necrotic intramural mass was identified in the uterine corpus. The tumor was classified as a cellular leiomyoma. RNA sequencing identified a *KAT6B*-*KANSL1* fusion that was confirmed by RT-PCR and Sanger sequencing. After 6 months of follow-up, the patient remains asymptomatic without evidence of disease.

**Conclusion:**

Prior studies of uterine leiomyomas have identified *KAT6B (*previously *MORF*) rearrangements in uterine leiomyomas, but this case is the first to identify a *KAT6B-KANSL1* gene fusion in a uterine leiomyoma. While alterations of *MED12* and *HMGA2* are most common in uterine leiomyomas, a range of other genetic pathways have been described. Our case contributes to the evolving molecular landscape of uterine leiomyomas.

## Background

Uterine leiomyomas are benign smooth muscle tumors found in nearly 70% of women by age 50. These tumors can cause significant symptoms related to heavy menstrual bleeding and pelvic pressure, leading many women to pursue hysterectomy. After menopause, almost all women will have a reduction in both size and number of leiomyomas [1]. Conversely, uterine sarcomas classically present as a rapidly enlarging pelvic mass with or without vaginal bleeding or pelvic pain and are more commonly found in postmenopausal women [2]. The clinical distinction of uterine leiomyoma from sarcoma is difficult and definitive diagnosis is possible only by pathologic examination. We present an unusual case of a rapidly enlarging uterine mass in a postmenopausal patient that, following hysterectomy, was classified as a cellular leiomyoma. Subsequent molecular genetic and cytogenetic analysis of the tumor identified a *KAT6B*-*KANSL1* gene fusion. The only prior reporting of this fusion transcript was a retroperitoneal leiomyoma that morphologically and genetically resembled a uterine leiomyoma [3].

## Case presentation

**Fig. 1:**
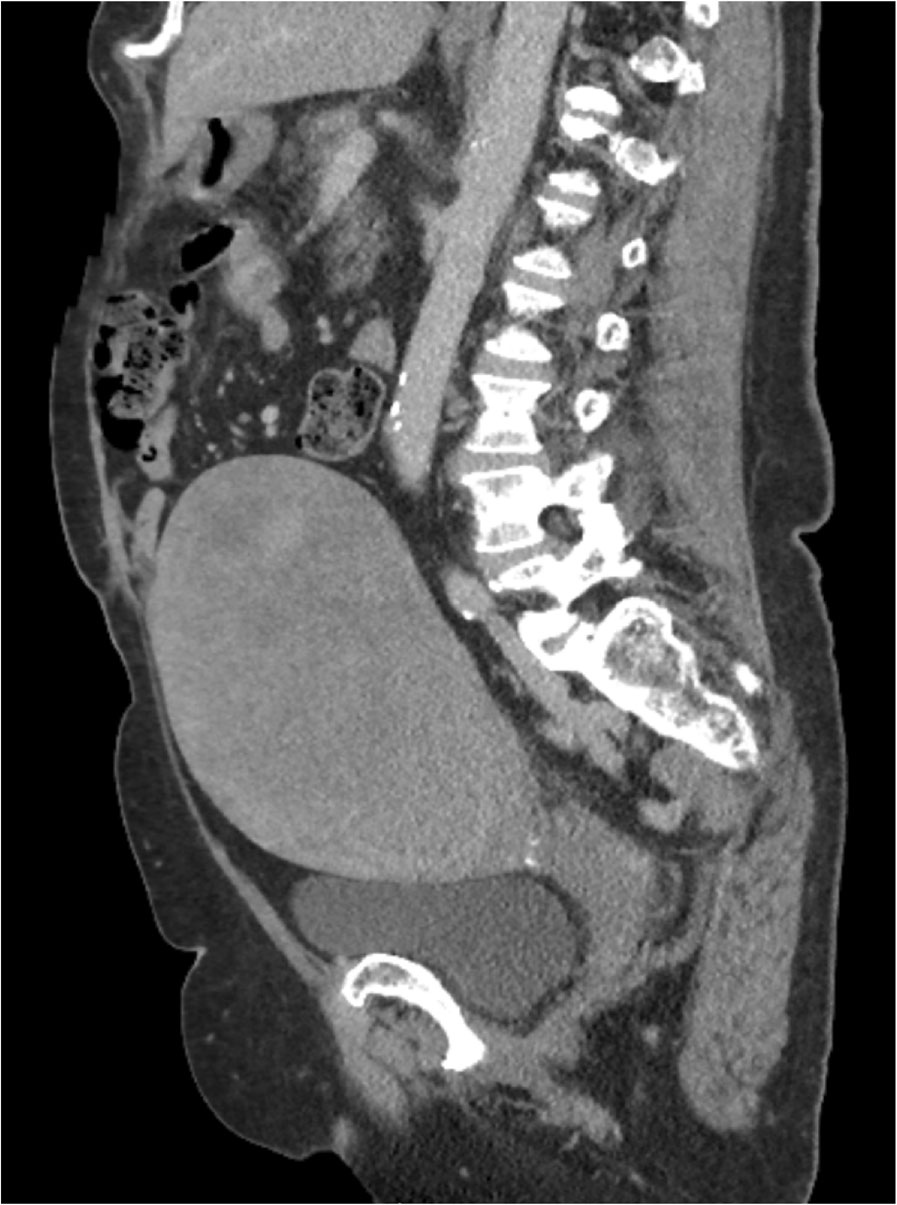
CT pelvis showing the large necrotic uterine mass

A 74 year-old Caucasian multiparous female with a history of ductal carcinoma in situ (DCIS) of the breast presented with significant postmenopausal bleeding, requiring blood transfusion, and a rapidly enlarging pelvic mass. DCIS was diagnosed 6 months prior to presentation and treated by unilateral total mastectomy and Tamoxifen which was transitioned to anastrozole at the onset of postmenopausal bleeding. She had a known history of uterine leiomyomas and no family history of uterine malignancy. Abdominal imaging by ultrasound and computerized tomography (CT) revealed a 15 cm heterogeneous mass located centrally within the anterior mid body of the uterus (Fig. 1). The patient underwent total abdominal hysterectomy and bilateral salpingo-oophorectomy.

**Fig. 2:**
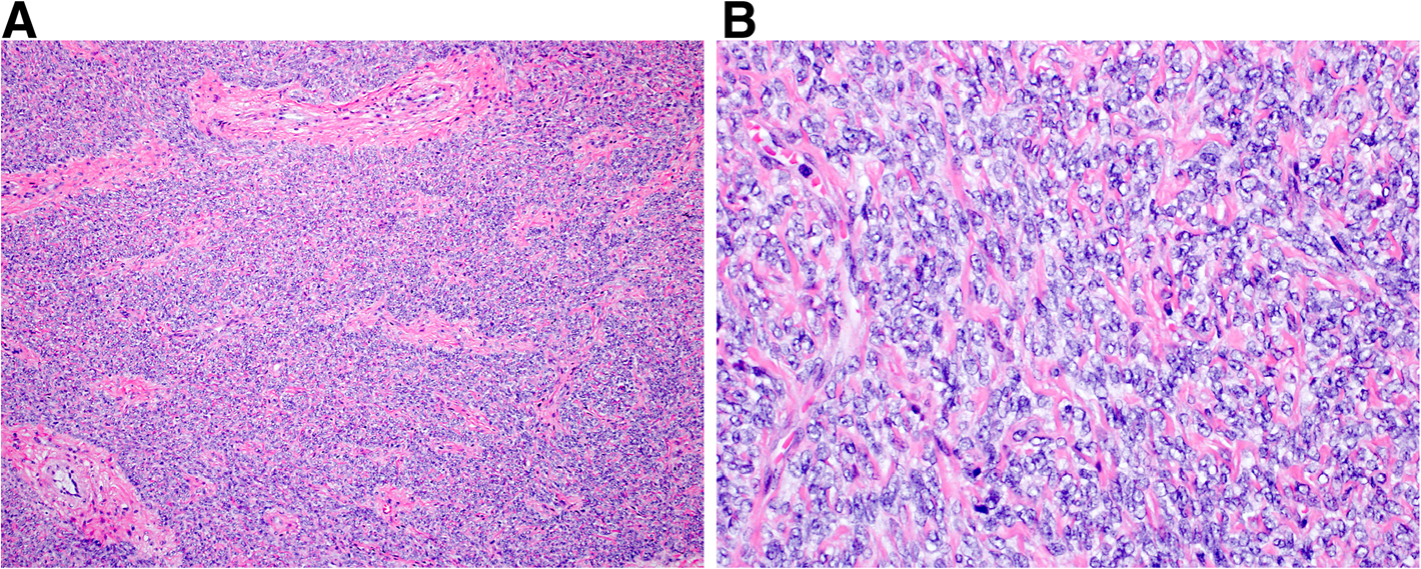
Hematoxylin and eosin photomicrographs of the large uterine cellular leiomyoma. The tumor formed anastomosing fascicles with intermixed thick-walled blood vessels and small fibrous zones (**a**, 100x). The cells had uniformly mild cytologic atypia with eosinophilic cytoplasm, round to ovoid nuclei with fine chromatin and small nucleoli (**b**, 400x)

The uterus with attached bilateral fallopian tubes and ovaries weighed 635 g. Grossly, the uterus contained a 15.5 cm well-demarcated intramural mass. The cut surface was white-tan to yellow with regions of necrosis. One section per centimeter of tumor was evaluated. Microscopically, the tumor was a cellular spindle cell neoplasm with anastomosing fascicles interrupted by thick-walled blood vessels or fibrous regions (Fig. 2a). A wispy or delicate hyaline extracellular matrix was seen throughout the tumor (Fig. 2b). The tumor cells had moderate eosinophilic cytoplasm, round to ovoid nuclei with fine chromatin and small nucleoli. The cells exhibited uniformly mild cytologic atypia. The mitotic index was no greater than 2 figures per 10 high power fields. Ischemic/hyaline-type necrosis was present, but no evidence of tumor cell/coagulative necrosis was identified. Immunohistochemically, the tumor strongly and diffusely expressed desmin and h-caldesmon and exhibited patchy, strong expression of CD10. Aside from two benign endometrial polyps, the remainder of the specimen was unremarkable. The intramural mass was classified as a cellular leiomyoma. The patient’s post-operative course was uneventful, and six months after surgery she remains asymptomatic without recurrence. She elected to discontinue all hormonal modulator therapy.

At the time of grossing, a sample of tumor was submitted for chromosomal karyotyping and RNA sequencing (RNA-Seq) according to a previously described protocol [4]. In brief, mRNA isolation, cDNA synthesis and library preparation utilized Illumina TruSeq Library Preparation Kit version 2 according to the manufacturer’s protocol. Sequencing was performed by the Illumina HiSeq 2500. A customized bioinformatics pipeline for RNA-Seq analysis known as MAP-RSeq was used to assess fusions and gene expression [5]. RNA-Seq gene expression analysis compared gene expression by the tumor to normal uterine tissue from the Genotype-Tissue Expression (GTEX) database (https://www.gtexportal.org/home/).

**Fig. 3:**
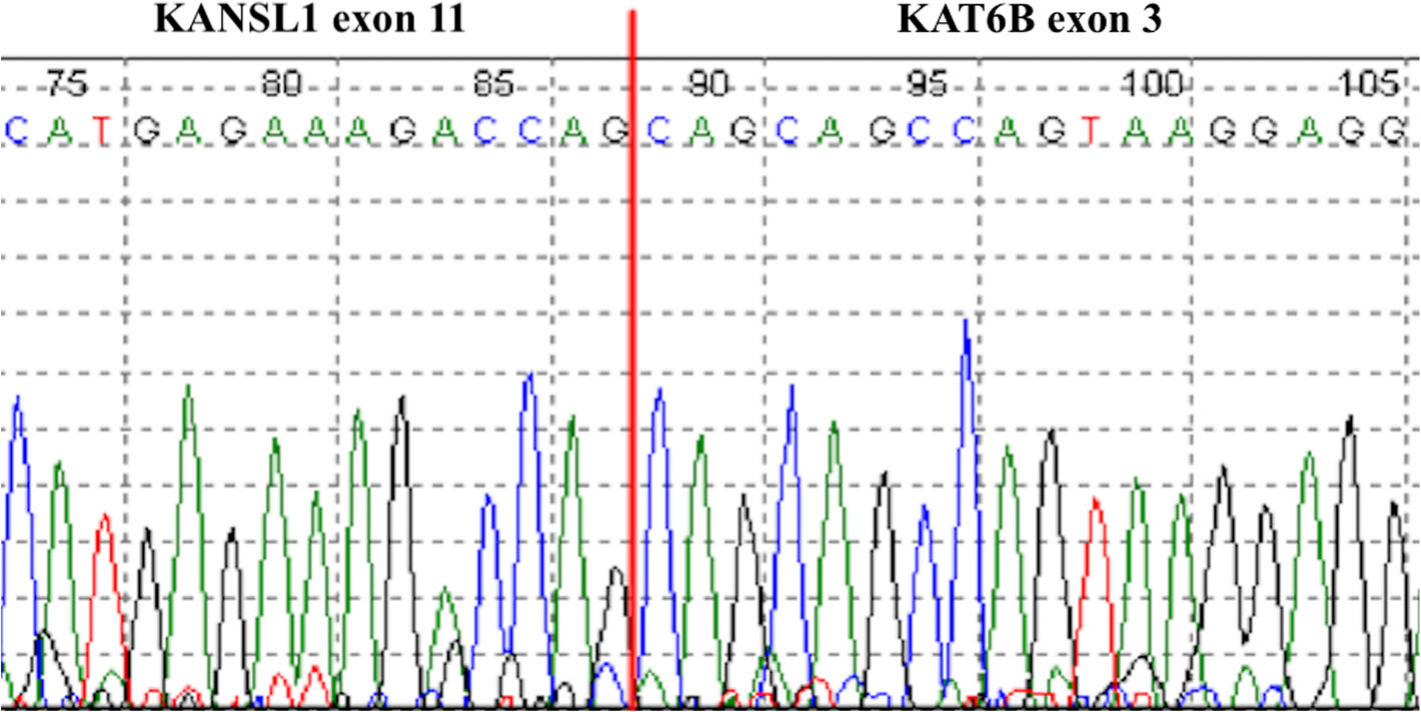
Sanger sequencing confirmation (reverse direction) of *KAT6B-KANSL1* sequence consistent with fusion of exon 3 of *KAT6B* to exon 11 of *KANSL1*

**Fig. 4:**
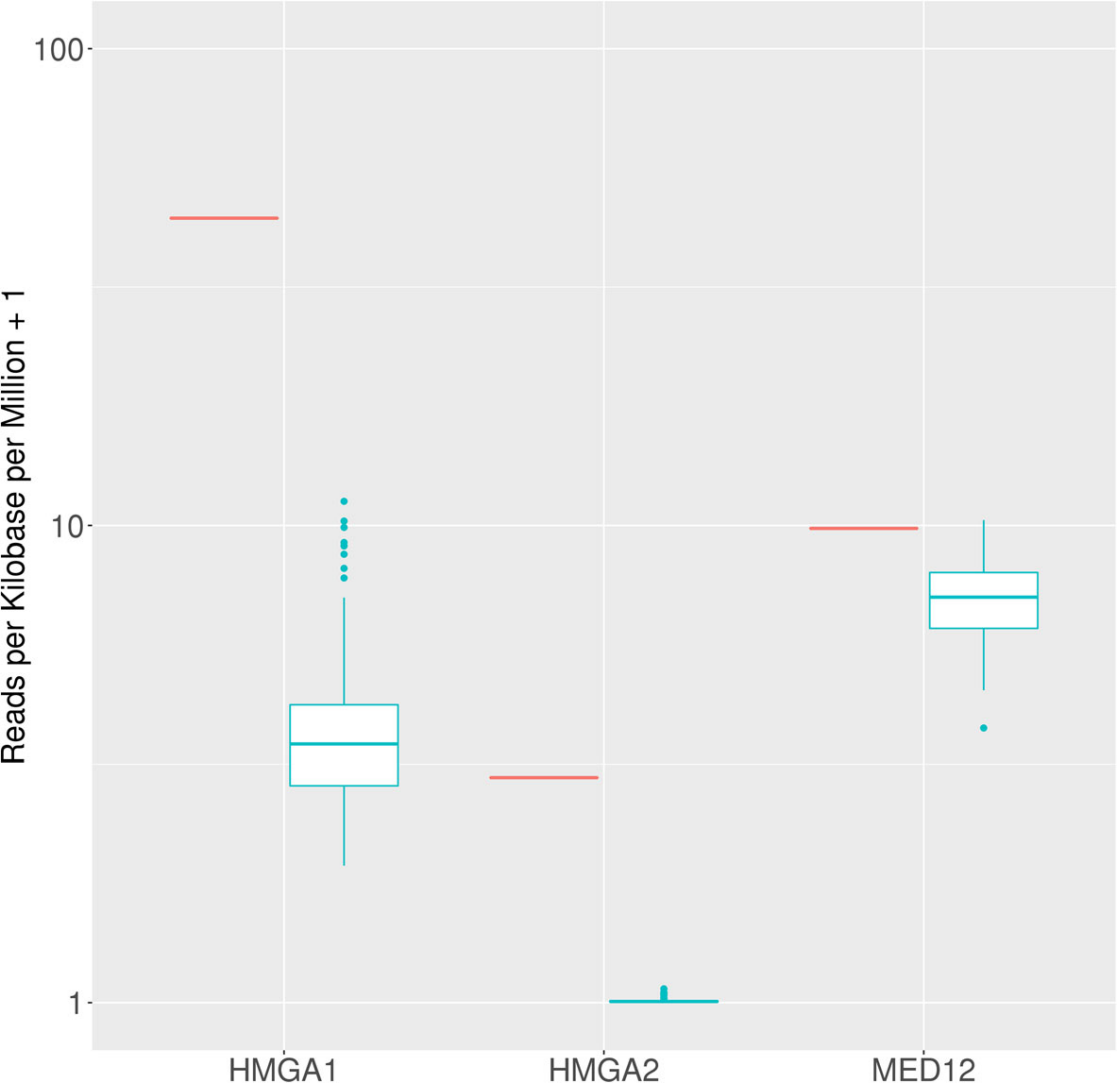
Levels of expression (normalized as Reads per Kilobase per Million) of the case (red color) compared to normal tissue values (uterus) obtained from GTEx with a logarithmic scale on the y-axis. *HMGA2* and *HMGA1* are overexpressed compared to the normal range, while *MED12* is within normal expression values

A conventional karyotype showed complex chromosomal abnormalities, which included numerous structural and numeric abnormalities: 45–46, X,der(3)t(1;3)(q21;q26), t(5;15)(q31;q22) add (6)(q25), ins(10;?)(q22;?),ins (11)(p15q21q23),+ 0-1mar[cp20]. RNA-Seq revealed a gene fusion involving *KAT6B* (10q22.2) and *KANSL1* (17q21.31). The fusion joined exon 3 of *KAT6B* to exon 11 of *KANSL1*. RT-PCR generated the expected fragment size of 165 bp and Sanger sequencing confirmed the fusion (Fig. 3). Gene expression analysis of *MED12*, *HMGA1* and *HMGA2* revealed overexpression of *HMGA2* and *HMGA1* and normal expression of *MED12* relative to normal uterine tissue (Fig. 4).

## Discussion

Alterations of mediator complex subunit 12 (*MED12*) and high mobility group AT-hook 2 (*HMGA2*) are the most common genetic aberrations in uterine leiomyomas [6, 7]. *MED12* is part of a multiprotein complex that acts to phosphorylate the C-terminal of RNA polymerase II. Missense mutations and in-frame insertion–deletions of *MED12*, frequently involving exon 2, disrupt the interaction of this complex and lead to diminished activity of a cyclin-dependent kinase and therefore decreased inhibition of transcription [6]. Aberrant function of MED12 has been shown to account for 70% of uterine leiomyomas [6, 7]. *HMGA2* is an oncogene and functions through a cell cycle checkpoint in the G1/S phase [8]. Tumors with *HMGA2* translocations represent larger, solitary lesions compared to those with *MED12* mutations [9].

Until recently, *MED12* and *HMGA2* were thought to be mutually exclusive genetic events since no uterine leiomyomas had been found to harbor both alterations. *HMGA2* translocations were described in 45% of tumors not containing *MED12* mutations [10], suggesting multiple, less prevalent, genetic aberrations are responsible for the remaining 55% of non-*MED12* altered leiomyomas. However, a study by Galindo et al. found multiple leiomyomas containing both *MED12* and *HMGA2* alterations also had the greatest degree of complex chromosomal rearrangements [11]. The authors proposed that *HMGA2* overexpression may be a consequence of these rearrangements [10, 11].

Monocytic leukemia zinc finger protein-related factor (*MORF*) located at 10q22, now known as *KAT6B*, is involved in histone acetyltransferase and allows segments of DNA to be more accessible to transcription factors. Moore and colleagues [12] previously described rearrangement of *MORF* in four uterine leiomyomas. In three tumors, they also mapped a break involving 17q21 but did not identify a gene fusion partner. The fusion of *KAT6B* and *KANSL1* (the latter positioned at 17q21) has been identified only once previously in a retroperitoneal leiomyoma [3]. It has been proposed that *KAT6B*-*KANSL1* might function in regulation of transcription [3]. Although *KAT6B* rearrangements have been reported in leiomyomas, rare examples of uterine leiomyosarcoma [13] and acute myeloid leukemia [3, 12], fusions involving *KANSL1* are rare. However, the three leiomyomas described by Moore et al. with a 17q21 rearrangement likely are tumors with *KAT6B-KANSL1* fusion [12]. A study of 9 uterine leiomyomas by Ozisik and investigators also identified 10q22 as a site of translocation in 8 typical leiomyomas and 1 cellular leiomyoma [14].

Recent advances in the genetics of leiomyoma tumorigenesis have proposed various pathways by which to categorize these tumors. A recent review organized genotypic and phenotypic alterations into categories of constitutional variants, somatic alterations and epigenetic mechanisms [15]. Constitutional variants refer to hereditary tumor susceptibility syndromes, including hereditary leiomyomatosis and renal cell carcinoma (HLRCC) and Alport Syndrome. Somatic alterations allow for classification by molecular subtype, broadly stratifying into structural abnormalities and mutations. Lastly, epigenetic mechanisms describe gene expression influences such as methylation. Another study that characterized the genetic events underlying uterine leiomyomas by means of whole genome sequencing and gene expression analysis uncovered four distinctive pathways, ranging from oncogenic stress from *MED12* aberrations, metabolic stress from inactivation of *FH* to complex chromosomal rearrangements induced by chromothripsis-like events and a variety of simple gene rearrangements [16].

Our case is the first to identify a *KAT6B-KANSL1* gene fusion in a uterine leiomyoma, a finding that contributes to the evolving molecular landscape of leiomyomas that may allow for tailored therapeutic intervention or development of targeted therapy.

## Abbreviations

DCIS: Ductal carcinoma in situ
CT: Computerized tomography
MORF: Monocytic leukemia zinc finger protein-related factor
MED12: mediator complex subunit 12
HMGA2: high mobility group AT-hook 2
